# Immunotherapy-Induced Hypophysitis Following Treatment With Tislelizumab in an Elderly Patient With Bladder Cancer and Prostate Cancer: A Case Report

**DOI:** 10.7759/cureus.51015

**Published:** 2023-12-23

**Authors:** Ning Zhang, Xuan Qu, Xiaochen Zhang, Xiaohong Sun, Lin Kang

**Affiliations:** 1 Department of Geriatrics, Peking Union Medical College Hospital, Peking Union Medical College, Chinese Academy of Medical Sciences, Beijing, CHN; 2 Department of Geriatrics, Peking Union Medical College, Chinese Academy of Medical Sciences, Beijing, CHN

**Keywords:** treatment, programmed cell death protein 1 inhibitors, immune checkpoint inhibitors, autoimmune hypophysitis, pituitary disease

## Abstract

Immune checkpoint inhibitors represent a hopeful and emerging group of medications employed in the regulation of the immune response against cancer, displaying tremendous potential in cancer treatment. However, the administration of these drugs has been linked to the occurrence of adverse events, among which hypophysitis appears to be a prevailing complication affecting a substantial number of patients. Given the potential gravity of this condition, it is strongly recommended to actively monitor hormone levels throughout the treatment process, allowing for the prompt detection and provision of appropriate therapeutic measures. The present study showcases a case involving a 72-year-old individual afflicted with both bladder cancer and prostate cancer, who subsequently developed autoimmune hypophysitis and secondary adrenocortical insufficiency following the administration of programmed death protein 1 (PD-1) inhibitors.

## Introduction

Cancer cells possess the capability to masquerade as regular cells by augmenting the expression of particular controlling receptors of the immune system [1]. This grants them the ability to elude identification and elimination by T cells, allowing them to propagate and multiply without detection from the immune system. Medications called immune checkpoint inhibitors (ICIs) aim to target these cancer cells by obstructing their specific receptors, thereby triggering T cells to assault the cancer cells. Over time, ICIs have transformed cancer treatment[2]. One form of ICI, named programmed death protein 1 (PD 1) inhibitors, functions by invigorating the body's immune response against tumors [3]. These anti-tumor medications have effectively treated diverse forms of cancer in certain patients. Nonetheless, there has been a mounting apprehension regarding immune-related adverse events (irAEs) connected with PD 1 inhibitors. These unfavorable events can result in immune reactions restricted to particular organs or tissues, impacting the gastrointestinal tract, liver, lungs, skin, endocrine system, and more [4]. One such significant reaction that cannot be brushed aside is ICIs-induced hypophysitis (IH). Timely identification of autoimmune hypophysitis occurring during the use of ICIs is crucial, as failure to do so promptly can lead to a pituitary crisis, which may even pose a life-threatening situation[5]. This article describes a case study involving an elderly patient with bladder cancer and concomitant prostate cancer who developed hypophysitis following the use of PD-1 inhibitors.

## Case presentation

The patient, a 72-year-old male, presented with persistent hematuria for one year, accompanied by nausea and vomiting four months prior to presentation. On June 6, 2022, the patient experienced gross hematuria without any identifiable triggers, along with frequent urination, urination urgency, and difficulty in urination. An enhanced abdominal and pelvic CT scan conducted at a local hospital revealed the presence of multiple cauliflower-like tumors in the bladder. The larger tumors, measuring approximately 3.1×2.2 cm, were found on the right side wall, suggesting a possible diagnosis of bladder cancer. Additionally, abnormal signals were observed in the prostate, indicating potential prostate cancer. On August 29, 2022, the patient underwent transurethral resection of bladder tumors and cystoscopy. During the procedure, multiple cauliflower-like tumors were discovered on the right wall, triangular area, and roof wall of the bladder. The larger tumors, measuring approximately 3.2×2.5 cm, were primarily located on the right wall, while the remaining tumors had a diameter of about 0.5-1.5 cm. The surgical team successfully removed the tumors and a 0.5 cm margin of surrounding tissue, reaching the bladder muscle layer. The postoperative pathology revealed a low-grade urothelial carcinoma of the bladder, classified as T1N0M0 stage bladder cancer. Following the surgery, the patient received gemcitabine 1,000 mg/m^2^ on day one and day eight, repeated every 21 days for a total of two courses of chemotherapy. On October 19, 2022, a second transurethral resection of the bladder tumor was performed at the local hospital, and the postoperative pathology did not reveal any urothelial cancer. At the same time, a transrectal ultrasound-guided prostate biopsy was conducted, which showed the presence of prostate adenocarcinoma (Gleason 4+3=7, Grade Group 2) in the prostate tissue. The stage of prostate cancer is T2N0M0. Starting from October 29, 2022, the patient received tislelizumab (200 mg) every three weeks, for a total of five doses, with the last dose administered on January 21, 2023. Additionally, the patient underwent endocrine therapy with abiraterone 1,000 mg qd, combined with goserelin 3.6 mg every four weeks, along with intravesical chemotherapy using gemcitabine 1,000 mg once a week, for a total of seven cycles of chemotherapy. Since late February 2023, the patient has been experiencing worsening nausea and vomiting. This has progressed from vomiting once every two to three days to vomiting stomach contents half an hour after eating each day. However, the patient did not show any signs of headache, vision impairment, or polyuria. A gastroscopy conducted at a local hospital diagnosed the patient with chronic atrophic gastritis. Treatment involved acid suppression and peripheral fluid rehydration, but the symptoms did not improve. Torso 18 F-FDG PET/CT showed no abnormal uptake suspicious of malignancy or inflammation. The patient was then admitted to multiple hospitals and received parenteral nutrition support and antiemetic treatment. However, his condition did not improve significantly, and his symptoms continued to worsen. In order to seek further diagnosis and treatment, the patient was admitted to the Geriatrics Department of Peking Union Medical College Hospital on June 12, 2023. Over the past four months, the patient had experienced a significant loss of appetite, accompanied by fatigue, a notable decline in physical function, and a weight loss of 19 kg. The patient's past medical history is significant for hypertension for which he has been taking amlodipine. There is no noteworthy personal history, marriage and childbearing history, or family history. Upon admission, the patient had a body temperature of 36.5°C, a pulse rate of 66 beats/min, a respiration rate of 20 breaths/min, and a blood pressure of 100/72 mmHg. His body weight was 44 kg, and his BMI was 15.9 kg/m^2^. It is worth noting that the patient was classified as frail based on the Fried criteria. The physical examination of the heart, lungs, and abdomen did not reveal any obvious abnormalities. There was no edema observed in both lower limbs. The patient exhibited normal physiological reflexes, but no pathological reflexes were elicited.

**Figure 1:**
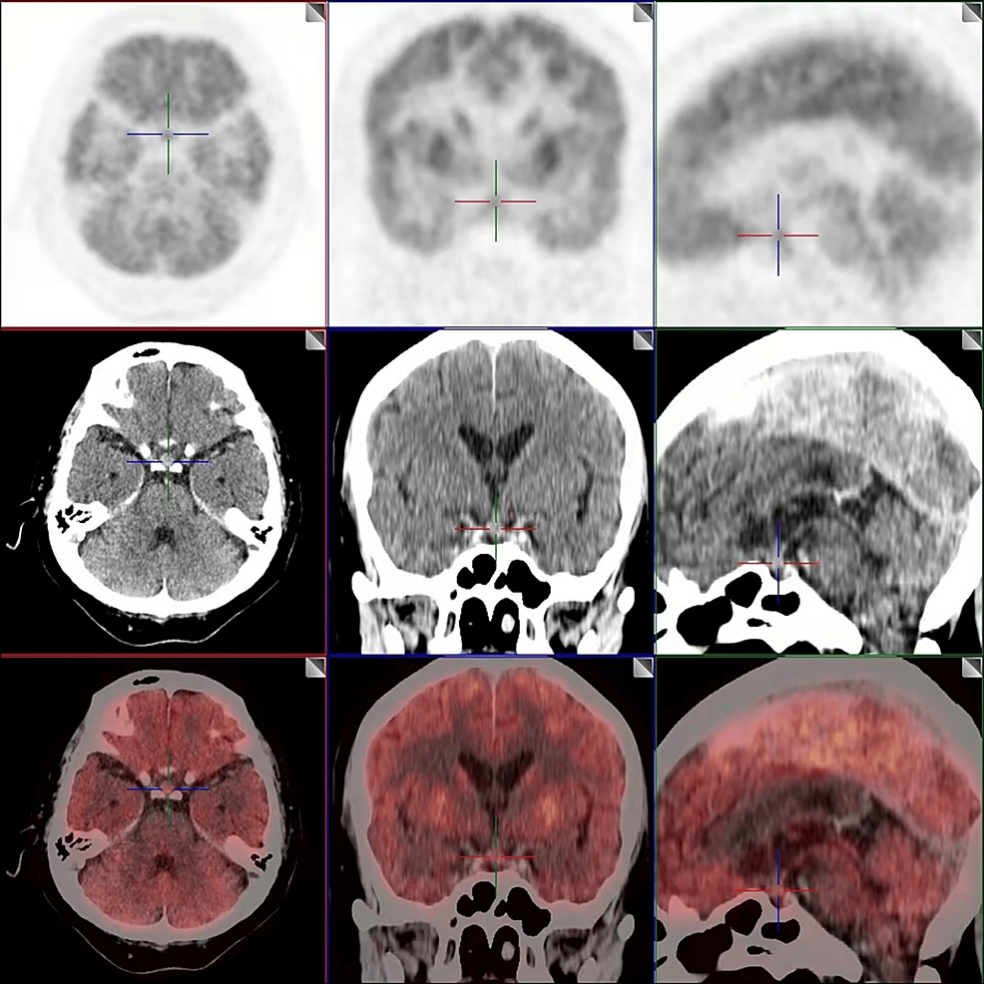
Brain PET/CT imaging of the patient indicated thickening of the pituitary stalk with increased radioactive uptake, along with slightly increased uneven radioactive uptake of the pituitary gland.

The results obtained after the tests are shown in Table 1. Analysis of the anterior pituitary function indicated hypoadrenocortical function and testicular hypofunction. Additionally, primary hypothyroidism was detected. Gastrointestinal tract angiography did not reveal any apparent obstruction. The routine magnetic resonance imaging (MRI) examination of the head showed fullness of the pituitary gland with uneven signal. Brain PET/CT imaging indicated thickening of the pituitary stalk with increased radioactive uptake (SUVmax 6.4), along with slightly increased uneven radioactive uptake of the pituitary gland (SUVmax 5.9) (Figure 1). Based on the patient's medical history and examination results, it was considered that PD-1 inhibitors were responsible for the development of autoimmune hypophysitis, secondary adrenocortical insufficiency, and testicular hypofunction. The patient's symptoms, such as nausea, vomiting, and fatigue, were gradually relieved with a daily intravenous infusion of 50 mg of hydrocortisone. After seven days, the dosage was adjusted to 20 mg of oral hydrocortisone in the morning and 20 mg in the afternoon for ongoing maintenance treatment. Additionally, the patient received oral levothyroxine at a dose of 12.5 μg/d for hypothyroidism and was prescribed oral nutrition supplements (ONS) daily. Two months after discharge, follow-up assessments on October 24, 2023, revealed improved adrenocortical function with ACTH levels of 8.2 pg/mL at 8:00, serum cortisol levels of 7.8 μg/dL, and 24-hour urinary free cortisol levels of 36.2 μg/24 hr. In addition, thyroid function at follow-up revealed a thyroid stimulating hormone (TSH) level of 104.874 μIU/mL, free thyroxine (FT4) level of 2.25 ng/dL, and free triiodothyronine (FT3) level of 1.57 pg/mL. The patient's serum sodium, albumin, and prealbumin levels were within normal ranges. Additionally, his body weight has increased by 5 kg, resulting in a BMI of 17.8 kg/m^2^. The patient is currently undergoing regular follow-up in the outpatient clinic.

**Table 1:** Laboratories of the patient during his hospital stay.

	Values (Reference values)
White blood cells (10^9^/L)	9.41（3.50 to 9.50）
Red blood cell count (10^12^/L)	3.28（4.00 to 5.50）
Hemoglobin (g/L)	104↓（120 to 160）
Hematocrit (%)	30.3↓ (35.0 to 50.0）
Platelets (10^9^/L)	318 (100 to 350）
Sodium (mmol/L)	130↓ (135 to 145）
Chloride (mmol/L)	89↓ (96 to 111）
Potassium (mmol/L)	3.6 (3.5 to 5.5）
Calcium (mmol/L)	2.01 (2.13 to 2.70）
Fasting blood glucose (mmol/L)	4.4 (3.9 to 6.1）
Albumin (g/L)	33↓ (35 to 52）
Prealbumin (mg/L)	159↓ (200 to 400）
Phosphorus (mmol/L)	1.40 (0.81 to 1.45）
Creatinine (μmol/L)	53 (59 to 104）
Urea (mmol/L)	5.10 (2.78 to 7.14）
Growth hormone (ng/mL)	1.8 (<2.0 ng/mL)
Insulin-like growth factor (μg/L)	71 (35 to 216)
Luteinizing hormone (IU/L	4.79 (1.24 to 8.62)
Follicle-stimulating hormone (IU/L)	11.51 (1.27 to 19.26)
Estradiol (ng/L)	19 (11 to 44)
Testosterone (ng/mL)	1.64↓(1.75 to 7.81)
Prolactin (ng/mL)	44.2↑ (2.6 to 13.1)
Thyroid-stimulating hormone (μIU/mL)	140.481↑ (0.380 to 4.340)
Free Thyroxine (FT4) (ng/dL)	0.13↓ (0.81 to 1.89)
Free triiodothyronine (FT3) (pg/mL)	1.29↓ (1.80 to 4.10)
Total thyroxine (TT4) (μg/dL)	1.80↓ (4.30 to 12.50)
Total triiodothyronine (TT3) (ng/mL)	0.52↓(0.66 to1.92)
Serum cortisol levels (μg/dL)	<0.50↓ (4.0 to 22.3)
Adrenocorticotropic hormone (ACTH) (pg/mL)	<3.00↓ (7.2 to 63.3)
24-hour urinary free cortisol (μg/24hr)	11.8↓ (12.3 to 103.5)

## Discussion

This case report discusses the development of autoimmune hypophysitis in an elderly patient with bladder cancer and prostate cancer. The patient had been treated with tislelizumab one month before and experienced rare side effects, including hypoadrenocortical insufficiency and testicular hypofunction. Tislelizumab is an anti-PD-1 monoclonal antibody that exhibits a distinct mechanism of binding to PD-1, which distinguishes it from other PD-1 inhibitors such as pembrolizumab and nivolumab[6]. Currently, the available clinical data on tislelizumab is limited. However, it has demonstrated encouraging outcomes in various clinical trials focused on the treatment of advanced NSCLC[7]. There is very limited literature on pituitary injury associated with tislelizumab. In a case study, a 71-year-old male patient diagnosed with advanced NSCLC encountered multiple-organ irAEs encompassing the lung, muscle, myocardium, liver, and pituitary gland following a single treatment cycle lasting 21 days with tislelizumab [8]. 

Autoimmune hypophysitis, a rare inflammatory disorder affecting the pituitary gland, is characterized by a significant infiltration of lymphocytes and plasma cells [9]. The main clinical manifestations of this condition include pituitary enlargement and hypopituitarism. Autoimmune hypophysitis is classified into two types based on its etiology: primary hypophysitis and secondary hypophysitis. The use of ICIs, such as PD-1 inhibitors, in the treatment of non-small-cell lung cancer has been identified as one of the factors contributing to the development of autoimmune hypophysitis[10]. Diagnosing autoimmune hypophysitis is primarily presumptive, as obtaining a pathological diagnosis (considered the 'gold standard') is challenging, and reliable serum markers for pituitary/hypothalamic antibodies are currently unavailable[11].

Immunotherapy-induced hypophysitis (IH) is more prevalent in men than primary hypophysitis[12]. Risk factors for IH include being over the age of 50 and receiving high-dose ICIs[13]. The occurrence of IH is most prevalent when ICI combination therapy with anti-PD(L)1-anti-CTLA-4 is utilized (9%-10% prevalence), whereas treatment with anti-CTLA-4 alone leads to a lower incidence (2%-6%), and anti-PD-1 monotherapy results in the lowest occurrence (1%) [14]. Patients who receive treatment regimens that include the use of anti-CTLA-4 antibodies experience the onset of IH within the initial three to four months of therapy, whereas cases related to anti-PD-1 monotherapy typically manifest at a later point in treatment, with a median occurrence of six months[15]. Therefore, it is crucial to continuously monitor and assess related hormone levels. IH primarily presents as anterior pituitary hypofunction, with potential involvement of the posterior pituitary, and frequently leads to secondary adrenocortical hypofunction. Additionally, it can cause secondary hypothyroidism, hypogonadotropic hypogonadism, and central diabetes insipidus. Multiple studies indicate isolated ACTH deficiency resulting from IH, with PD-1 inhibitors being associated with a higher occurrence of isolated ACTH deficiency compared to CTLA-4 inhibitors [16]. Regarding imaging, approximately 50% of IH patients display pituitary MRI abnormalities about one week before the clinical diagnosis of hypopituitarism. Pituitary shrinkage generally occurs between four to 12 weeks later [17]. Pituitary enlargement is a common characteristic observed in pituitary MRI scans of IH patients [18]. Some studies have summarized the progression of IH and suggest that early inflammation is manifested as pituitary enlargement, while pituitary atrophy and vacuolated sella turcica may ultimately ensue [19]. In general, the MRI manifestations of IH in the pituitary gland are comparable to those of primary hypophysitis. Nonetheless, IH may exhibit mild or temporary enlargement of the pituitary gland, and occasionally, the pituitary MRI may fail to identify any abnormal indications. Approximately 23% of IH patients demonstrate normal results in their pituitary MRI, whereas only 2% of primary hypophysitis patients present normal pituitary MRI [18]. Thus, it is insufficient to exclude IH solely based on normal pituitary MRI findings. Enhanced pituitary MRI reveals diffuse enlargement of the pituitary gland in more than 50% of patients with hypophysitis[20]. Therefore, it is crucial to suggest dynamic observation of pituitary-enhanced MRI both before and during the administration of ICIs to ensure accurate monitoring.

Despite the growing recognition of ICI-related hypophysitis, there is still much that is not understood about its biological background. This lack of understanding is a reflection of our incomplete knowledge of the biological background of irAEs. Currently, the prevailing hypothesis for explaining the pathophysiology of irAEs suggests that there is a complex interaction involving cellular autoimmunity, humoral immunity, cytokines, chemokines, and genetics [21]. Additionally, the precise mechanism of tislelizumab-induced hypophysitis remains unclear. Furthermore, while the overexpression of PD-1L in functional pituitary adenomas has been observed due to genetic alterations or an inflammatory environment, the expression of PD-1L and/or PD-1 in normal human pituitary tissue remains unknown[22].

In 2021 and 2022, the American Society of Clinical Oncology (ASCO) and the European Society for Medical Oncology (ESMO) issued clinical practice guidelines addressing the management of immunotherapy toxicity [23,24]. Based on these guidelines, severe toxicity necessitates the termination of ICI therapy, except in cases of hormonal control of endocrine disorders. The first-line treatment for IH typically entails the use of high-dose glucocorticoid (prednisone, approximately 1 mg/kg). However, despite an initial positive response to this therapy, the overall failure and recurrence rates are substantial [25]. A study conducted by Min et al. [26] demonstrated that systemic glucocorticoid treatment does not improve the prognosis of pituitary function. Current understanding suggests that the majority of pituitary damage caused by ICIs is irreversible, necessitating long-term hormone replacement therapy for most patients with ICI-induced hypophysitis. This holds particularly true for patients who develop hypophysitis as a result of PD-1/PD-1L and CTLA-4 inhibitors, where no distinction exists among patients [27]. Based on the current proposal, it is suggested that continuing ICI treatment would be advantageous for patients with Grade 1-2 IH. This is because the treatment helps sustain the therapeutic impact on the primary tumor. Conversely, it is recommended to discontinue ICI treatment for patients with Grade 3-4 IH. This is due to the potential risk of multiple pituitary crises associated with the continuation of treatment in these patients[28].

## Conclusions

In our case, the patient's clinical symptoms, which included fatigue, anorexia, nausea, and vomiting, were unusual and could be misdiagnosed as worsening of the primary tumor or intracranial metastasis. To eliminate other potential causes, diagnostic tests such as digestive tract angiography and brain MRI were conducted. Further examination revealed secondary adrenocortical hypofunction, and other conditions such as pituitary tumor and immune-induced hypophysitis were progressively ruled out. The possibility of PD 1 inhibitors causing autoimmune hypophysitis and secondary adrenocortical insufficiency was eventually considered. Symptoms of ICI-related hypophysitis can be subtle or overlap with symptoms of tumor diseases, particularly fatigue and headache, which are often ignored. It is recommended to regularly monitor endocrine hormones, such as thyroid hormone and cortisol, before and during the use of ICIs in order to identify potential drug-immune-mediated endocrine lesions and prevent pituitary crises. Early identification of rare adverse drug reactions and prompt treatment are vital for clinicians.
